# Regulation of *Drosophila* Metamorphosis by Xenobiotic Response Regulators

**DOI:** 10.1371/journal.pgen.1003263

**Published:** 2013-02-07

**Authors:** Huai Deng, Tom K. Kerppola

**Affiliations:** Department of Biological Chemistry, University of Michigan Medical School, Ann Arbor, Michigan, United States of America; University of California San Francisco, United States of America

## Abstract

Mammalian Nrf2-Keap1 and the homologous *Drosophila* CncC-dKeap1 protein complexes regulate both transcriptional responses to xenobiotic compounds as well as native cellular and developmental processes. The relationships between the functions of these proteins in xenobiotic responses and in development were unknown. We investigated the genes regulated by CncC and dKeap1 during development and the signal transduction pathways that modulate their functions. CncC and dKeap1 were enriched within the nuclei in many tissues, in contrast to the reported cytoplasmic localization of Keap1 and Nrf2 in cultured mammalian cells. CncC and dKeap1 occupied ecdysone-regulated early puffs on polytene chromosomes. Depletion of either CncC or dKeap1 in salivary glands selectively reduced early puff gene transcription. CncC and dKeap1 depletion in the prothoracic gland as well as *cncC^K6/K6^* and *dKeap1^EY5/EY5^* loss of function mutations in embryos reduced ecdysone-biosynthetic gene transcription. In contrast, dKeap1 depletion and the *dKeap1^EY5/EY5^* loss of function mutation enhanced xenobiotic response gene transcription in larvae and embryos, respectively. Depletion of CncC or dKeap1 in the prothoracic gland delayed pupation by decreasing larval ecdysteroid levels. CncC depletion suppressed the premature pupation and developmental arrest caused by constitutive Ras signaling in the prothoracic gland; conversely, constitutive Ras signaling altered the loci occupied by CncC on polytene chromosomes and activated transcription of genes at these loci. The effects of CncC and dKeap1 on both ecdysone-biosynthetic and ecdysone-regulated gene transcription, and the roles of CncC in Ras signaling in the prothoracic gland, establish the functions of these proteins in the neuroendocrine axis that coordinates insect metamorphosis.

## Author Summary

Human Nrf2-Keap1 and the fruit fly CncC-dKeap1 protein complexes function both in response to foreign chemicals and in development. We found that CncC and dKeap1 control fruit fly development by regulating the production and actions of the principal hormone that controls the transformation of larvae into pupae. In hormone-responsive cells, CncC and dKeap1 bound to the genes that are activated by the hormone. When the amount of CncC or dKeap1 in these cells was reduced, the genes were not activated efficiently. When the amount of CncC or dKeap1 was reduced in the organ where the hormone is made, the genes whose products make the hormone were not activated efficiently. Because less hormone was made, it took longer for the larvae to turn into pupae, and the resulting pupae were bigger. Reduction of the amount of CncC intercepted previously identified signals for pupation. Nrf2 is required for the same signals to cause cancer in mice. The effects of CncC and dKeap1 both on genes that control hormone production and on genes that are switched on by the hormone in different organs indicate that they have multiple roles in the transformation of fruit fly larvae into pupae.

## Introduction

Cellular responses to many xenobiotic compounds, including various toxins and pharmacological agents, are controlled by mammalian Nrf2 and Keap1, and by the homologous *Drosophila* CncC and dKeap1 proteins [1], [2], [3]. The Nrf2-Keap1 complex has multiple effects on carcinogenesis. Nrf2-deficient mice have increased susceptibility to chemical carcinogens, potentially because of defective activation of cytoprotective genes in response to carcinogen exposure [4]. Mutations in Nrf2 and Keap1 that are predicted to disrupt their interactions are found in many human cancers, suggesting that Nrf2 interactions with Keap1 counteract cancer progression [1], [5]. Conversely, the deletion of Nrf2 suppresses pancreatic and lung tumorigenesis in a mouse model with constitutively active K-Ras^G12D^ expression [6]. The mechanisms whereby Nrf2 promotes tumorigenesis in conjunction with K-Ras^G12D^ are not known. Nrf2 and Keap1 are investigated as potential targets for therapeutic interventions in cancer, neurodegenerative diseases and developmental disorders [1], [7].

Nrf2 (NF-E2-Related Factor 2) is a bZIP family transcription factor that can bind to genes whose transcription is induced by xenobiotic compounds [1]. Keap1 (Kelch-like ECH-Associated Protein 1) is a Kelch family protein that can interact with the N-terminal region of Nrf2, and inhibits the activation of many genes activated by Nrf2 [8]. Studies in cultured mammalian cells indicate that Keap1 is predominantly localized to the cytoplasm [9], where it promotes Nrf2 degradation and inhibits its accumulation in the nucleus [8], [10], [11], [12].

**Figure 1:**
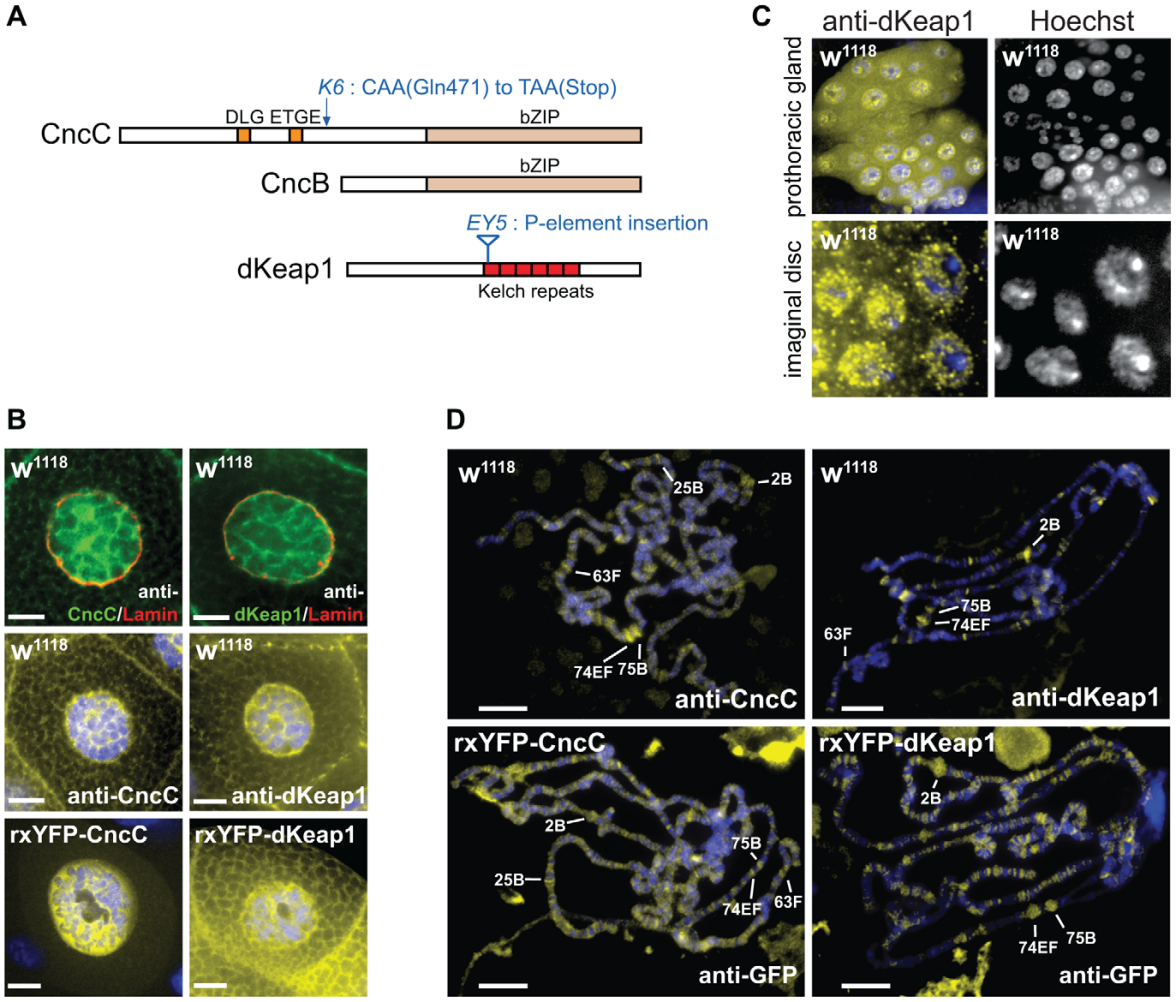
CncC and dKeap1 subcellular localization and polytene chromosome occupancy. (A). Conserved sequences and mutations in CncC, CncB and dKeap1. Regions that are conserved between *Drosophila* and mammalian proteins are shown in color. The locations of the *cnc^K6^* and *dKeap1^EY5^* mutations are indicated [3], [15]. (B). Visualization of the subcellular distributions of CncC and dKeap1 in salivary glands. [Upper panels] Endogenous CncC and dKeap1 distributions in wild type (*w^1118^*) salivary glands stained using anti-CncC or anti-dKeap1 (green) and anti-Lamin Dm0 (red) antibodies. [Middle panels] Wild type (*w^1118^*) salivary glands were stained with anti-CncC or anti-dKeap1 antibodies (yellow) and Hoechst (blue). *[Lower panels]* Ectopic CncC and dKeap1 distributions in live salivary glands that expressed rxYFP-CncC and rxYFP-dKeap1, respectively. The intrinsic fluorescence (yellow) was superimposed on Hoechst fluorescence (blue). The salivary glands were isolated from early wandering 3^rd^ instar larvae. The scale bars are 10 µm. (C). Visualization of the localization of endogenous dKeap1 in the prothoracic gland and in imaginal disc cells. Endogenous dKeap1 was visualized by immunostaining using anti-dKeap1 antibodies (yellow) superimposed on Hoechst staining (blue). The separate images are shown to the right of each color image. (D). Visualization of the loci occupied by CncC and dKeap1 on polytene chromosomes. [Upper panels] The loci occupied by endogenous CncC and dKeap1 were visualized on polytene chromosome from wild type larvae (*w^1118^*) by staining with anti-CncC and anti-dKeap1 antibodies, respectively. [Lower panels] The loci occupied by ectopic CncC and dKeap1 were visualized on polytene chromosomes from larvae that expressed rxYFP-CncC and rxYFP-dKeap1, respectively, by staining with anti-GFP antibodies. The immunofluorescence (yellow) was superimposed on Hoechst fluorescence (blue). The major ecdysone-regulated early puffs are indicated. The polytene chromosomes were prepared from the salivary glands of early wandering 3^rd^ instar larvae.

Studies of the *Drosophila* homologues of Nrf2 and Keap1 have provided insights into the functions of these protein families in adult flies. The *Drosophila cap‘n’collar* locus encodes CncC, which contains a bZIP domain homologous to that of Nrf2 and N-terminal DLG and ETGE motifs homologous to those that mediate Nrf2 interaction with Keap1 [13] (Figure 1A). *Drosophila* dKeap1 contains Kelch repeats homologous to those that mediate Keap1 interaction with Nrf2 as well as a sequence motif that is required for mammalian Keap1 export from the nucleus [3], [10]. Overexpression of CncC and depletion of dKeap1 in adult flies activates the transcription of many genes that protect cells from xenobiotic compounds, whereas dKeap1 overexpression represses their transcription, indicating that the functions of these protein families in the xenobiotic response are conserved between mammals and *Drosophila* [2], [3].

Several lines of evidence suggest that CncC and dKeap1 also affect cell proliferation and development. CncC overexpression and dKeap1 depletion inhibit intestinal stem cell proliferation, and counteract the proliferative effects of environmental stress in these cells [14]. Loss of function mutations in *cncC* and *dKeap1* cause larval lethality [3], [15]. The genes regulated by CncC and dKeap1 during larval development had not been established. Elucidation of the relationship between CncC and dKeap1 functions in xenobiotic responses and in development is important to define how the transcription regulatory functions of CncC and dKeap1 are regulated in response to intrinsic and extrinsic stimuli.

In *Drosophila* and in other holometabolous insects, the onset of metamorphosis is triggered by an increase in the level of the endocrine hormone ecdysone [16], [17]. Ecdysone is synthesized in the prothoracic gland (PG) by a series of cytochrome P450 enzymes [18]. The expression of these ecdysone-biosynthetic genes and the timing of pupation are regulated by Ras signaling in response to prothoracicotropic hormone (PTTH) binding to the Torso receptor [19], [20]. Ecdysone facilitates the onset of metamorphosis by regulating transcription in many tissues, including the salivary glands where ecdysone-regulated transcription is manifest by puffs at specific polytene chromosome loci [21]. The transcription factors that bind to the ecdysone biosynthetic gene promoters and activate their transcription have remained unknown.

In the work presented here, we found that CncC and dKeap1 occupied the classical ecdysone-regulated puffs on polytene chromosomes. Depletion of CncC or of dKeap1 in salivary glands reduced ecdysone-regulated gene transcription. Depletion of CncC or of dKeap1 in the PG as well as *cncC* and *dKeap1* loss of function mutations reduced ecdysone biosynthetic gene transcription in larvae and in embryos, respectively. The reduced ecdysteroid levels caused by CncC and by dKeap1 depletion in the PG delayed pupation and suppressed the premature pupation caused by constitutive Ras signaling. These observations establish roles for CncC and dKeap1 in transcriptional programs in different tissues that coordinate metamorphosis.

## Results

### Nuclear localization of CncC and dKeap1

To investigate if the subcellular localization of CncC was regulated by dKeap1 in the manner that has been reported for mammalian Nrf2 and Keap1, we determined the distributions of CncC and dKeap1. Both CncC and dKeap1 immunoreactivity were predominantly nuclear in *Drosophila* salivary gland cells (Figure 1B, Figure S1A). Likewise, ectopic CncC and dKeap1 fused to fluorescent proteins were enriched within the nuclei of live salivary gland cells (Figure 1B, Figure S1A). CncC and dKeap1 were also present in the nuclei of prothoracic gland, imaginal disc and gut cells, though the proportions that were localized to the nucleus varied in different tissues (Figure 1C, Figure S1B). The intensity of anti-dKeap1 immunoreactivity was markedly reduced in *dKeap1^EY5/EY5^* mutant larvae, and the bands corresponding to endogenous dKeap1 and CncC were not detected by immunoblotting of extracts from *dKeap1^EY5/EY5^* and *cnc^K6/K6^* mutant larvae, demonstrating the specificity of these antibodies (Figure S1C, S1D). These observations establish that both endogenous as well as ectopically expressed CncC and dKeap1 were localized to the nuclei in many different tissues, in contrast to the predominantly cytoplasmic localization observed for Keap1 and Nrf2 in many cultured mammalian cell lines.

### CncC and dKeap1 occupancy at ecdysone-regulated puffs on polytene chromatin

To establish if CncC and dKeap1 bound to specific chromatin loci, we visualized their occupancy on polytene chromosomes by immunostaining. Anti-CncC and anti-dKeap1 antibodies recognized overlapping sets of loci, including a majority of the classical ecdysone-regulated early puffs on polytene chromosomes (e.g. 2B, 74EF, 75B, 63F, and 25B) (Figure 1D). Anti-CncC antibodies also recognized several loci that were not detected by anti-dKeap1 antibodies (e.g. 22B and 97B) and vice versa (e.g. 50C and 94C). CncC and dKeap1 occupied many non-puff loci, and did not occupy all puffs, indicating that their occupancy was not controlled solely by chromatin decondensation. Ectopically expressed CncC and dKeap1 fusion proteins occupied loci that overlapped those occupied by endogenous CncC and dKeap1, though they also occupied additional loci (Figure 1D). Few other sequence-specific DNA binding proteins have been identified that bind to ecdysone-regulated puffs [22], [23], [24]. The overlapping sets of loci occupied by endogenous and ectopic CncC and dKeap1, as detected by several different antibodies, corroborate the specificity of CncC and dKeap1 binding at these loci.

### Regulation of ecdysone response genes by CncC and dKeap1 in salivary glands

**Figure 2:**
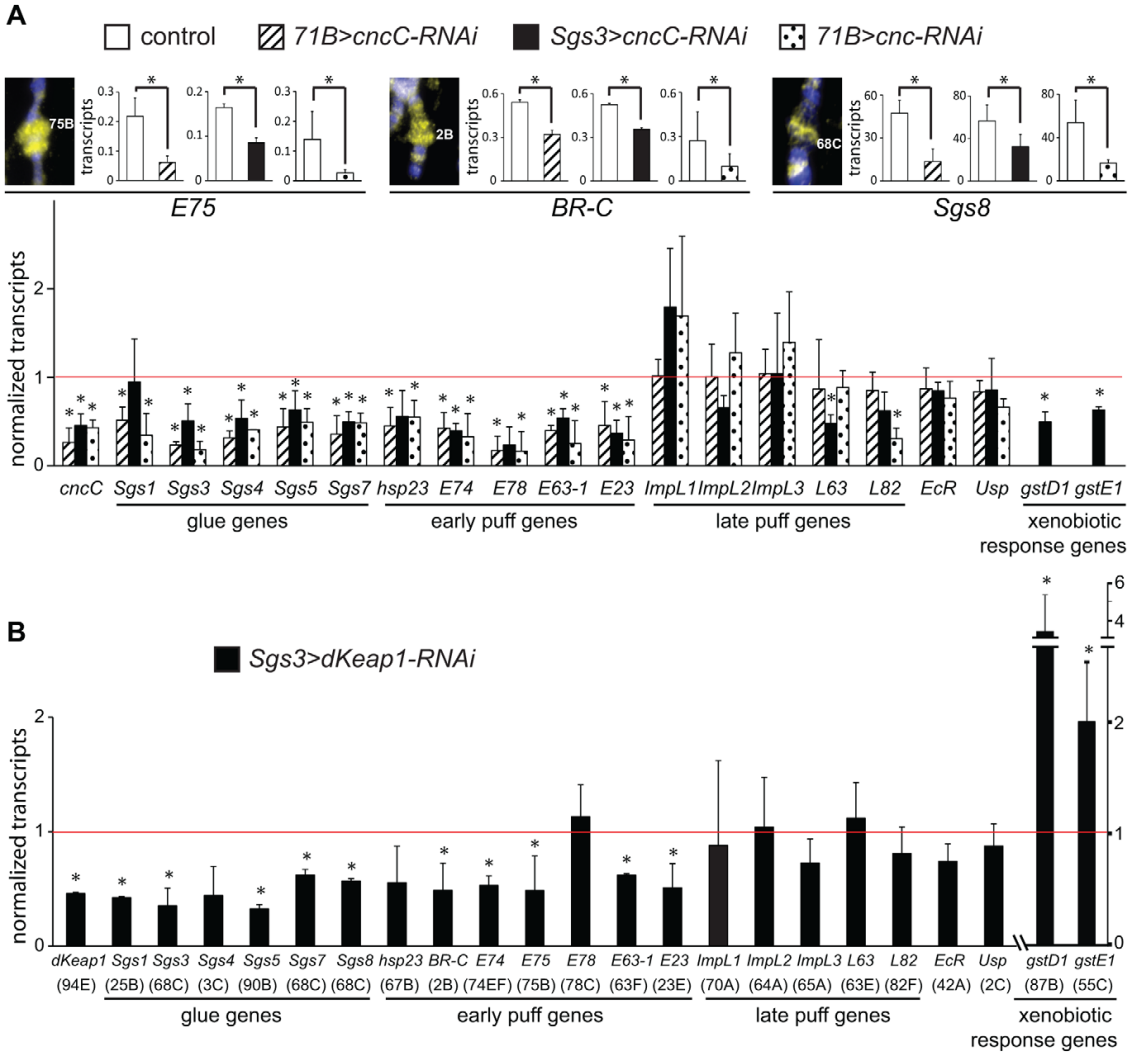
Regulation of early puff gene transcription by CncC and dKeap1. (A). Effects of CncC depletion in the salivary glands on the transcription of ecdysone-regulated genes and xenobiotic response genes. The images show wild type polytene chromosomes stained using anti-CncC antibodies. The levels of the transcripts indicated below the bar graphs were measured in the salivary glands of early wandering 3^rd^ instar larvae. Transcript levels were compared in control larvae [*71B-GAL4* (*71B>*), *Sgs3-GAL4* (*Sgs3>*); open bars] and in larvae that expressed the shRNA targeting CncC under the control of the *71B-GAL4* (*71B>cncC-RNAi*; solid bars) or the *Sgs3-GAL4* (*Sgs3>cncC-RNAi*; striped bars) driver, as well as in larvae that expressed a different shRNA targeting all Cnc isoforms under the control of the *71B-GAL4* driver (*71B*>*cnc-RNAi*; dotted bars). Larvae with different drivers and shRNAs were analyzed in separate experiments under different conditions (see methods). To facilitate comparison, the transcript levels in the lower graph were normalized by the levels of the transcripts in the control larvae. (B). Effects of dKeap1 depletion in the salivary glands on the transcription of ecdysone-regulated genes and xenobiotic response genes. The levels of the transcripts indicated below the bar graphs were measured in the salivary glands of early wandering 3^rd^ instar larvae that expressed the shRNA targeting dKeap1 under the control of the *Sgs3-GAL4* driver as described for the lower graph in panel A. The data in panels A and B represent the means and standard deviations from three or four separate experiments each (*, p<0.05). The corresponding loci are indicated in parentheses below the lower graph.

To test if CncC and dKeap1 regulated transcription of the early puff genes that they occupied on polytene chromosomes, we investigated the effects of CncC as well as dKeap1 depletion in salivary glands on transcription of ecdysone-regulated genes. Expression of an shRNA that targets CncC [3] under the control of either the *71B-GAL4* or the *Sgs3-GAL4* driver reduced the levels of almost all of the ecdysone-regulated early puff and glue gene transcripts examined (Figure 2A). In contrast, transcription of most of the late puff genes that were not prominently occupied by CncC or dKeap1 was not affected by CncC depletion (Figure 2A). *71B-GAL4* directs expression throughout salivary gland development and in imaginal discs [25]; Transcription directed by *Sgs3-GAL4* is detected only in late 3^rd^ instar salivary glands [22], establishing that the change in transcription of ecdysone-regulated genes was due to CncC depletion in salivary glands. Expression of a different shRNA that targets all Cnc isoforms also reduced the levels of all of the early puff and glue gene transcripts examined (Figure 2A). The *cncC-RNAi* transgene had no detectable effects on transcription in larvae that lacked a GAL4 driver (Figure S2A).

Expression of an shRNA that targets dKeap1 [3] under the control of the *Sgs3-GAL4* driver also reduced the levels of almost all of the ecdysone-regulated early puff and glue gene transcripts examined, but had no effect on most of the late puff gene transcripts (Figure 2B). In contrast to the concordant effects of CncC and dKeap1 depletion on ecdysone-regulated early puff gene transcription, CncC versus dKeap1 depletion had opposite effects on transcription of the *gstD1* and *gstE1* xenobiotic response genes (Figure 2A, 2B) [3], [26].

To examine if CncC and dKeap1 depletion affected early puff gene transcription through indirect mechanisms, we measured the levels of ecdysone receptor subunit transcripts and ecdysteroids. CncC and dKeap1 depletion in the salivary glands had no effect on the levels of the *ecdysone receptor* (*EcR*) or *ultraspiracle* (*usp*) transcripts in the salivary glands (Figure 2A, 2B). CncC depletion in the salivary glands also had no effect on the level of 20-hydroxyecdysone (20E) in the larvae (Figure S2B). There was no detectable effect on the size or the morphology of the salivary glands, or on the time of pupation. CncC and dKeap1 therefore likely regulated transcription of the ecdysone-regulated genes directly by binding to these loci.

### Regulation of ecdysone biosynthetic genes by CncC and dKeap1

**Figure 3:**
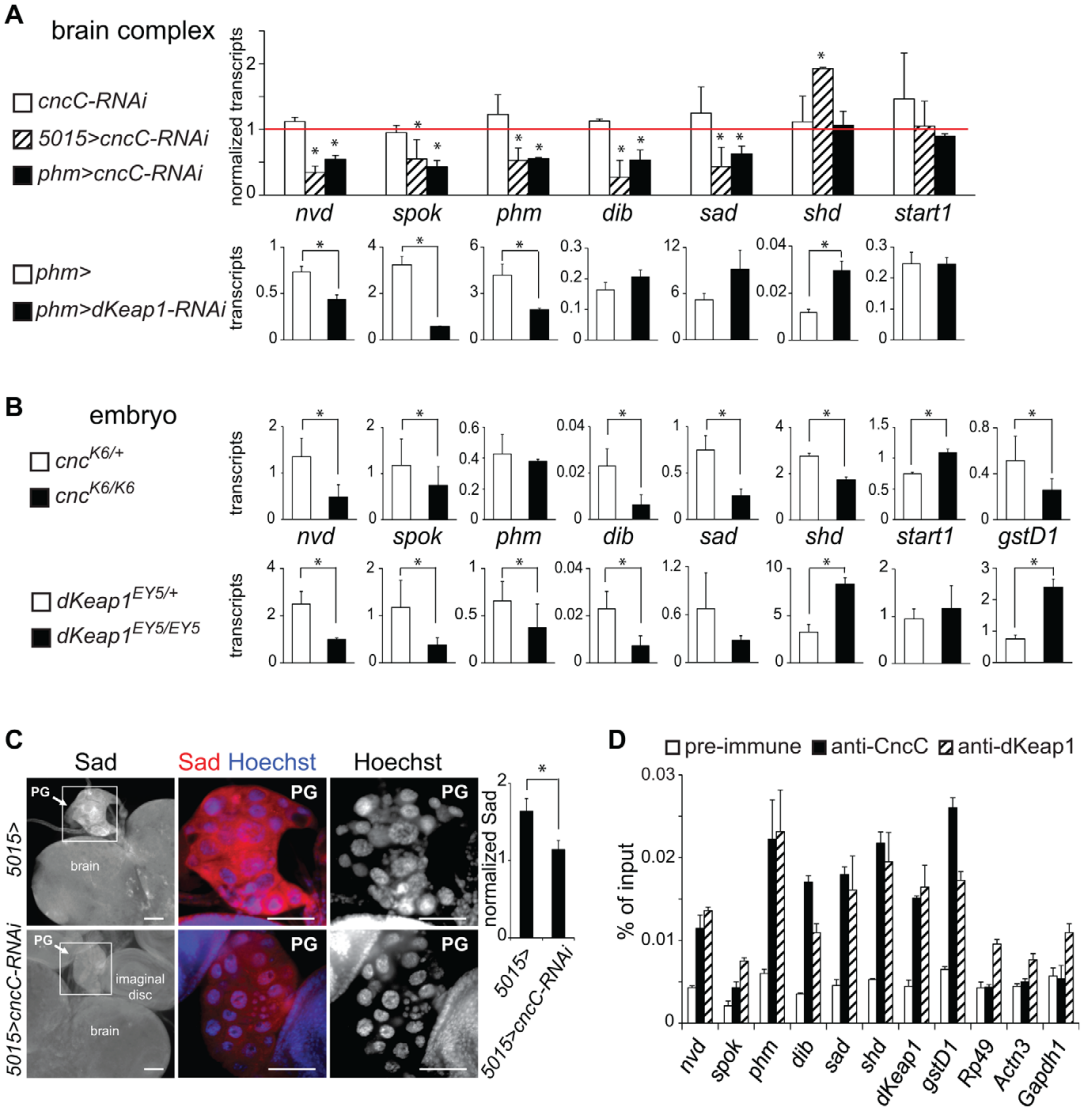
Regulation of ecdysone biosynthetic gene transcription by CncC and dKeap1. (A). Effects of CncC and dKeap1 depletion in the prothoracic gland on ecdysone biosynthetic gene transcription. [Upper graph] The transcripts indicated below the bars were measured in the brain complexes from early wandering 3^rd^ instar larvae that expressed the shRNA targeting CncC under the control of the *5015-GAL4* (*5015>cncC-RNAi*, striped bar) or the *phm-GAL4* (*phm>cncC-RNAi*, solid bar) driver, and from larvae that lacked a GAL4 driver (*cncC-RNAi*, open bar). To facilitate comparison, the transcript levels were normalized by the levels of the transcripts in control larvae (*5015>, phm>* and *w^1118^*). The data represent the means and standard deviations of the ratios of transcript levels between larvae that expressed the *cncC-RNAi* shRNA constructs and the corresponding control larvae from two independent experiments (*, p<0.05). [Lower graphs] The transcripts indicated above the graphs were measured in the brain complexes from early wandering 3^rd^ instar larvae that expressed the shRNA targeting dKeap1 under the control of the *phm-GAL4* (*phm>cncC-RNAi*, solid bar) driver, and from control larvae (*phm>*, open bar). The transcript levels were normalized by the levels of *Rp49* transcripts divided by 100 and represent the means and standard deviations from two separate experiments (*, p<0.05). (B). Effects of CncC and dKeap1 loss of function mutations on ecdysone biosynthetic gene and xenobiotic response gene transcription in late embryos. The transcripts indicated between the graphs were measured in heterozygous (*cnc^K6/+^* or *dKeap1^EY5/+^*, open bar) and homozygous (*cnc^K6/K6^* or *dKeap1^EY5/EY5^*, solid bar) stage 14–16 embryos. The transcript levels were normalized by the levels of *Rp49* transcripts divided by 1000 and represent the means and standard deviations from two separate experiments (*, p<0.05). (C). Effects of CncC depletion on PG morphology and Sad protein expression. The brain complexes of control larvae (*5015>*) and larvae that expressed the shRNA targeting CncC in the PG (*5015>cncC-RNAi*) were stained using anti-Sad (red) and Hoechst (blue). The ratio of Sad immunostaining in the PG relative to the brain is plotted in the graph to the right of the images (*, p<0.05). (D). Analysis of CncC and dKeap1 occupancy at the promoter regions of ecdysone biosynthetic genes in late embryos. Chromatin isolated from stage 14–16 embryos was precipitated using anti-CncC (solid bars), anti-dKeap1 (striped bars), and pre-immune (open bars) sera. The promoter regions of the genes indicated below the bars were quantified using qPCR. *gstD1* was used as a positive control and *Rp49, Actn3 a*nd *Gapdh1* were used as negative controls. The data represent the mean values and standard deviations of replicate qPCR reactions, and are representative of two experiments using independently prepared embryos.

The effects of CncC and dKeap1 on ecdysone-regulated gene transcription in salivary glands, the arrested development of *cnc^K6/K6^* and *dKeap1^EY5/EY5^* mutant larvae, and the presence of both CncC and dKeap1 in prothoracic gland nuclei prompted us to investigate their roles in ecdysone biosynthetic gene transcription. We investigated the effects of CncC and dKeap1 depletion in the prothoracic gland (PG) on ecdysone biosynthetic gene transcription. We measured the levels of the *neverland* (*nvd*), *spookie* (*spok*), *phantom* (*phm*), *disembodied* (*dib*), *shadow* (*sad*), and *shade* (*shd*) transcripts in the brain complexes of larvae that expressed the shRNA targeting CncC or dKeap1 in the PG. Expression of the shRNA targeting CncC under the control of either the *5015-GAL4* or the *phm-GAL4* driver reduced the levels of all ecdysone biosynthetic gene transcripts that are expressed exclusively in the PG (Figure 3A, Figure S3A). *5015-GAL4* directs expression in the PG, the salivary glands and the lymph gland [27]; *phm-GAL4* directs expression in the PG and at low levels in the wing and leg discs of 3^rd^ instar larvae [28]. Expression of the shRNA targeting CncC also reduced Sad immunoreactivity in the PG (Figure 3C, Figure S3B). Expression of the shRNA targeting dKeap1 under the control of the *phm-GAL4* driver reduced the levels of *nvd*, *spok*, *phm*, but not the levels of *dib* and *sad* in the brain complex (Figure 3A). CncC depletion in the PG therefore reduced transcription of all known ecdysone biosynthetic genes that are selectively expressed in the PG, and dKeap1 depletion reduced transcription of a subset of these genes.

To determine the specificity of the reduction in ecdysone biosynthetic gene transcription upon CncC or dKeap1 depletion in the PG, we examined transcription of *shd*, which is expressed throughout the brain, and *start1*, which is expressed predominantly in the PG [29]. The levels of *shd* and *start1* transcripts in the brain complex were not reduced by CncC or dKeap1 depletion in the PG (Figure 3A, Figure S3A). Expression of the shRNAs targeting CncC or dKeap1 also did not alter the size, morphology or the number of nuclei in the PG (Figure 3C, Figure S3B and S3C). It is therefore unlikely that the effects of CncC or dKeap1 depletion on ecdysone biosynthetic gene transcription were caused by a disruption of PG development.

To examine if CncC or dKeap1 affected ecdysone biosynthetic gene transcription at a different stage of development, we examined the effects of the *cnc^K6^* and *dKeap1^EY5^* loss of function mutations on transcription of these genes in late embryos. The levels of *nvd*, *spok*, *dib*, *sad*, and *shd* transcripts were lower in *cnc^K6/K6^* homozygous than in *cnc^K6/+^* heterozygous embryos (Figure 3B). Likewise, the levels of *nvd*, *spok*, *phm*, *dib*, and *sad* transcripts were lower in *dKeap1^EY5/EY5^* homozygous than in *dKeap1^EY5/+^* heterozygous embryos, whereas the level of *shd* transcripts was higher in the homozygous than in heterozygous embryos (Figure 3B). The moderate effects of the *cnc^K6^* and *dKeap1^EY5^* loss of function mutations on ecdysone biosynthetic gene transcription and the consequent lack of complete developmental arrest during embryogenesis could be due to maternal deposition of CncC and dKeap1 mRNA or proteins in the egg. The effects of these mutations on the levels of ecdysone biosynthetic gene transcripts in embryos corroborate the effects of CncC and dKeap1 depletion on transcription of these genes in the PG. In contrast to the concordant effects of the *cnc^K6^* and *dKeap1^EY5^* loss of function mutations on ecdysone biosynthetic gene transcription, these mutations had opposite effects on transcription of the *gstD1* xenobiotic response gene (Figure 3B). We were not able to determine the effects of CncC or dKeap1 depletion on the level of *gstD1* in the PG since *gstD1* is expressed throughout the brain.

The *cnc^K6^* and *dKeap1^EY5^* mutations could affect transcription of the ecdysone biosynthetic genes through several mechanisms, including direct binding to the promoters and indirect effects on other transcription factors. To test if CncC and dKeap1 bound to the ecdysone biosynthetic genes, we measured CncC and dKeap1 occupancy at their promoter regions in late embryos using ChIP analysis. CncC and dKeap1 occupancy were observed at the *phm*, *shd*, *dib* and *sad* genes at levels that were comparable to their occupancy at the *dKeap1* and *gstD1* genes (Figure 3D). Their occupancy was higher near the *sad* promoter compared to flanking regions (Figure S3D). No CncC occupancy above background and only low dKeap1 occupancy was observed at the *Rp49*, *Actn3*, and *Gapdh1* housekeeping genes. CncC and dKeap1 are therefore likely to regulate ecdysone biosynthetic gene expression directly by binding to their promoter regions.

### Effects of CncC and dKeap1 on ecdysteroid production and pupation

**Figure 4:**
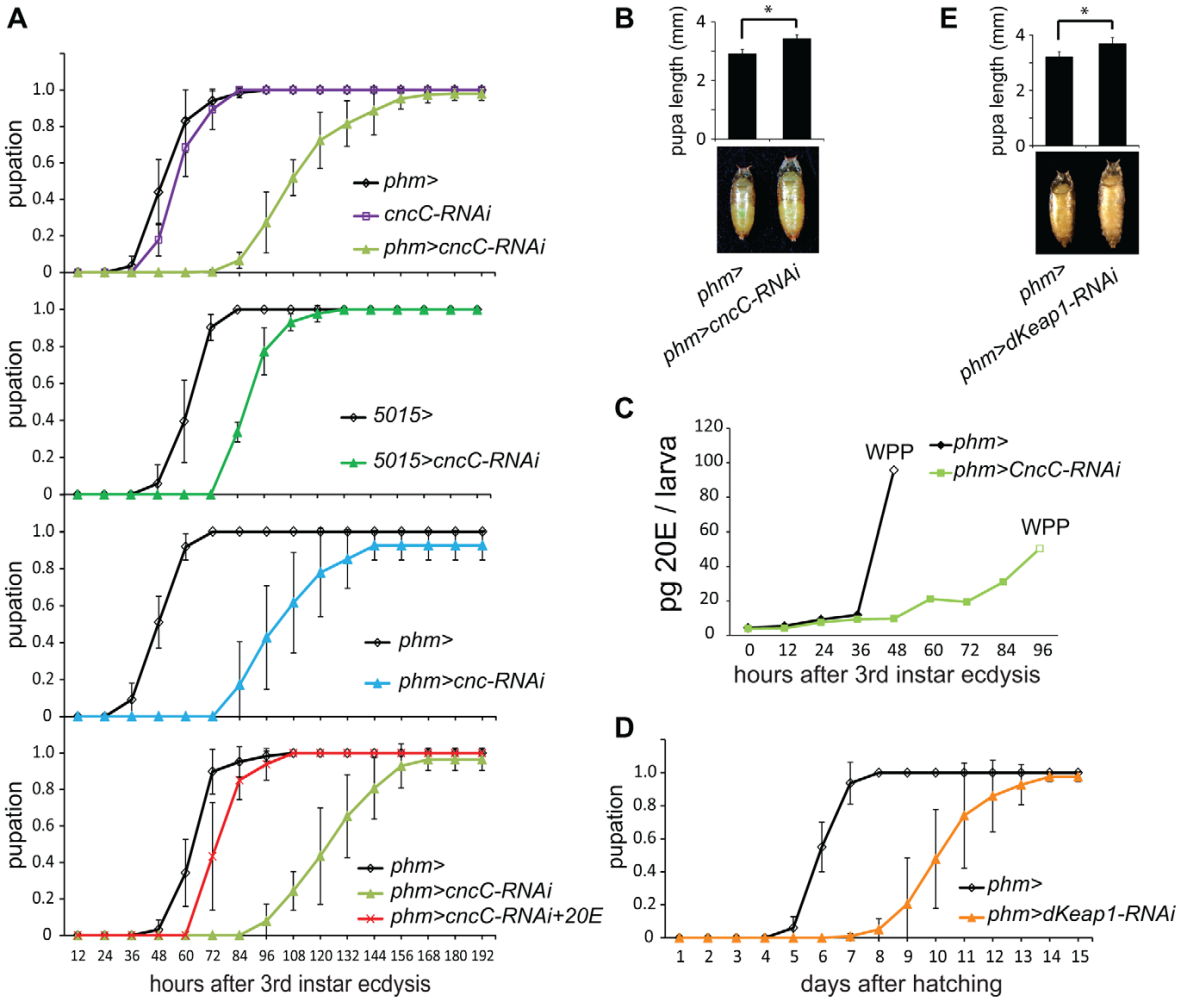
Effects of CncC and dKeap1 depletion on the time of pupation, pupal size, and ecdysteroid levels. (A). Effects of expression of the shRNA targeting CncC in the PG on the time of pupation. The proportion of larvae that had formed pupae was plotted as a function of the time after 3^rd^ instar ecdysis for control larvae (*phm>* or *5015>*, black; *cncC-RNAi*, purple), for larvae that expressed the shRNA targeting CncC (*phm>cncC-RNAi*, green; *5015>cncC-RNAi*, dark green), as well as for larvae that expressed a different shRNA targeting all Cnc isoforms (*phm>cnc-RNAi*, cyan) in the PG. Pupation by the larvae the expressed the shRNA targeting CncC was also examined when the food was supplemented with 0.5 mg/ml 20E (*phm>cncC-RNAi*+20E, red). The data in the different graphs were obtained from separate experiments and represent the means and standard deviations from two to four repeats using 20–30 larvae in each. (B). Effects of expression of the shRNA targeting CncC in PG on the sizes of pupae. The lengths of the pupae formed by control larvae (*phm>*) and larvae that expressed the shRNA targeting CncC in the PG (*phm>cncC-RNAi*) were measured. The data represent the mean and standard deviation of 30 pupae of each genotype (*, p<0.001). (C). Effects of expression of the shRNA targeting CncC in the PG on 20-hydroxyecdysone (20E) levels. The levels of 20E in larvae and white pre-pupae (WPP, open symbols) were measured in control (*phm>*, black) and transgenic *Drosophila* that expressed the shRNA targeting CncC in the PG (*phm>cncC-RNAi*, green). The 20E level at each time point was measured in 10 larvae or pre-pupae. (D). Effects of expression of the shRNA targeting dKeap1 in the PG on the time of pupation. The proportion of larvae that had formed pupae was plotted as a function of the time after hatching for control larvae (*phm>*, black) and for larvae that expressed the shRNA targeting dKeap1 (*phm>dKeap1-RNAi*, orange) in the PG. The data represent the means and standard deviations from four separate experiments using 20–30 larvae in each. The time of pupation was measured after hatching since it was more difficult to obtain a sufficient number of larvae synchronized at 3^rd^ instar ecdysis. Based on observation of a small number of larvae (>10), the time between 3^rd^ instar ecdysis and pupation was delayed by about 3 days upon dKeap1 depletion in the PG. The delay in pupation was therefore primarily due to extension of the 3^rd^ instar larval stage. (E). Effects of expression of the shRNA targeting dKeap1 in the PG on the sizes of pupae. The lengths of the pupae formed by control larvae (*phm>*) and larvae that expressed the shRNA targeting dKeap1 in the PG (*phm>dKeap1-RNAi*) were measured. The data represent the mean and standard deviation of 20 pupae of each genotype (*, p<0.001).

Defects in ecdysteroid biosynthesis in the PG can delay pupation and increase the size of the pupae [19]. We investigated if the reduction in ecdysone biosynthetic gene transcription caused by CncC or dKeap1 depletion affected the timing of pupation by altering larval ecdysteroid levels. Expression of the shRNA targeting CncC under the control of the *phm-GAL4* or the *5015-GAL4* driver extended the average time between third instar molting and pupation by 40–125% (Figure 4A). Expression of a different shRNA targeting all Cnc isoforms under the control of the *phm-GAL4* driver also delayed the time of pupation (Figure 4A). The *cncC-RNAi* transgene alone had no detectable effect. The mean size of the pupae formed by larvae that expressed the shRNA targeting CncC in the PG was larger than the mean size of the pupae formed by control larvae (Figure 4B), indicating that the delayed pupation was not a secondary consequence of a reduced rate of larval growth. Some larvae continued to grow and formed giant semi-pupae (Figure S4A).

To evaluate the role of ecdysteroid levels in the delayed pupation, we measured the level of 20E in the larvae. Expression of the shRNA targeting CncC in the PG delayed the rise in 20E after third instar molting (Figure 4C, Figure S4B). To establish if the reduced level of 20E was the cause of the delay in pupation, we added 20E to the food for the larvae that expressed the shRNA targeting CncC in the PG. Supplementation with 20E shortened the time between third instar molting and pupation in these larvae by almost 50%, restoring their time of pupation nearly to that of wild-type larvae (Figure 4A).

Expression of the shRNA targeting dKeap1 under the control of the *phm-GAL4* driver extended the average length of the larval stage by 4 days (Figure 4D). The mean size of the pupae formed by larvae that expressed the shRNA targeting dKeap1 in the PG was larger than the mean size of the pupae formed by control larvae (Figure 4E). Taken together, these results establish that CncC and dKeap1 affected the time of pupation through their effects on ecdysone biosynthetic gene transcription and on the level of 20E.

### Functions of CncC in response to Ras signaling

**Figure 5:**
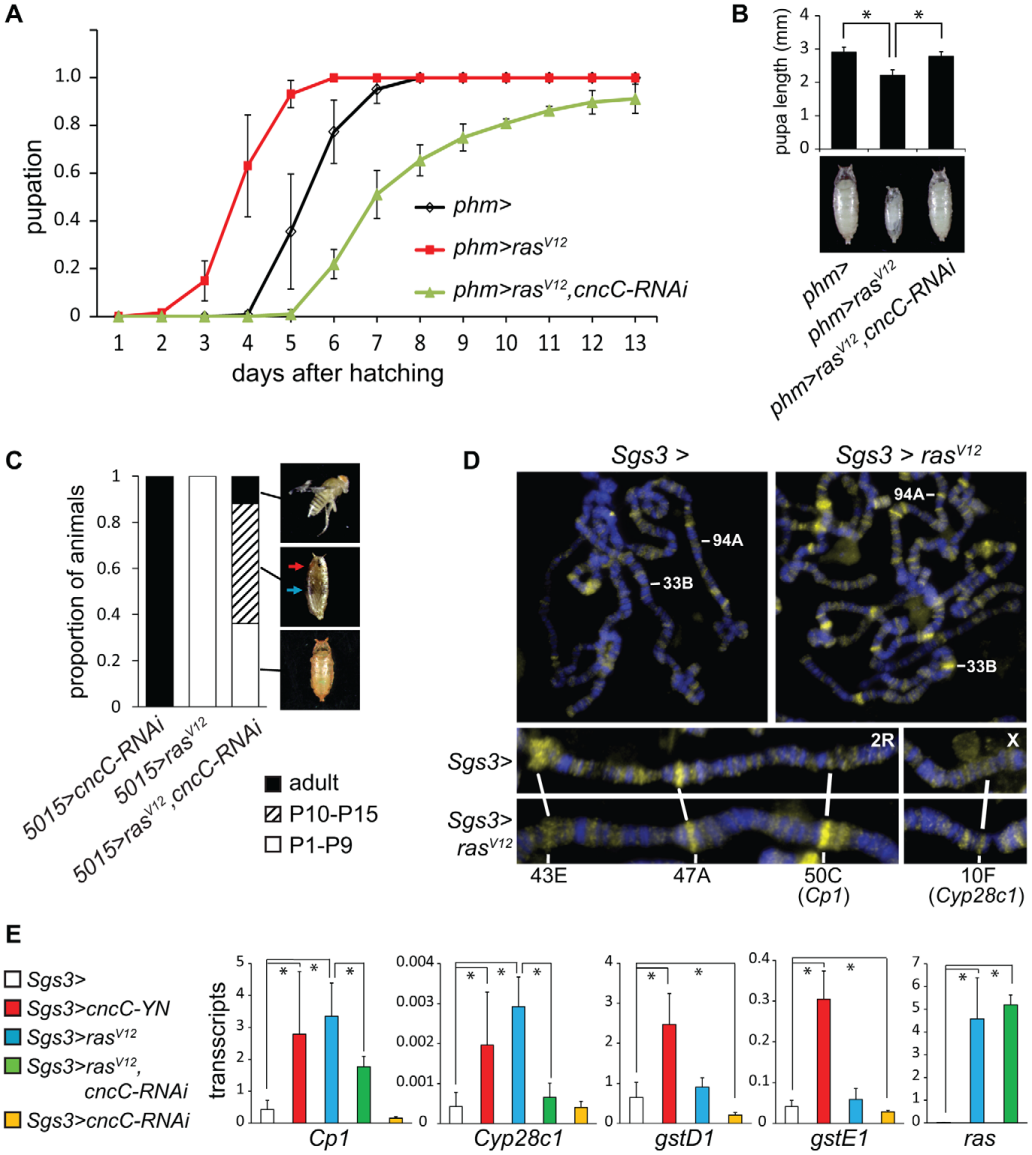
Interrelationships between Ras signaling and CncC functions. (A). Effects of CncC depletion on the time of pupation by larvae that expressed Ras^V12^ in the PG. The proportion of larvae that had formed pupae was plotted as a function of the time after hatching for control larvae (*phm>*, black), larvae that expressed either Ras^V12^ alone (*phm>ras^V12^*, red) or Ras^V12^ together with the shRNA targeting CncC (*phm>ras^V12^*, *cncC-RNAi*, green) in the PG. The data represent the means and standard deviations from three repeats using 20–30 larvae in each. (B). Effects of CncC depletion on the sizes of pupae formed by larvae that expressed Ras^V12^ in the PG. The lengths of pupae formed by the larvae described in part A were measured. The data represent the means and standard deviations of 30 pupae of each genotype (*, p<0.001). (C). Effects of CncC depletion and Ras^V12^ expression in the PG on pupal development. The terminal stage of development was recorded for pupae formed by larvae that expressed either the shRNA targeting CncC alone (*5015>cncC-RNAi*), Ras^V12^ alone (*5015>ras^V12^*), or Ras^V12^ in combination with the shRNA targeting CncC (*5015>ras^V12^*, *cncC-RNAi*) in the PG. The proportion of pupae that arrested at early (P1–P9, open bar) and late (P10–P15, striped bar) pupal stages and that eclosed (adult, solid bar) are indicated (images shown on the right). Early and late stage pupae were distinguished by the absence or presence of red eye pigment (red arrow) and dark wings (blue arrow). Approximately 100 animals of each genotype were scored. (D). Effects of Ras^V12^ expression in salivary glands on CncC occupancy on polytene chromosomes. Polytene chromosomes from control larvae (*Sgs3>*) and larvae that expressed Ras^V12^ (*Sgs3>ras^V12^*) were stained using anti-CncC antibody. Selected loci whose occupancy by CncC changed upon Ras^V12^ expression are labeled. (E). Effects of Ras^V12^ and CncC fusion or CncC-RNAi expression in salivary glands on transcription of genes whose occupancy was affected by Ras^V12^ expression and of control xenobiotic response genes. The levels of the transcripts indicated above the graphs were measured in salivary glands that expressed the proteins or the shRNA indicated. All transcript levels were normalized by the level of *Rp49* transcripts. The data represent the means and standard deviations from two separate experiments (*, p<0.05).

We examined the functions of CncC in relation to the Ras signaling pathway, which controls the timing of pupation in response to prothoracicotropic hormone (PTTH) binding to the Torso receptor [19]. Constitutively active Ras^V12^ expression in the PG causes early pupation and a smaller pupal size [20] (Figure 5A). Moreover, deletion of Nrf2 in mice suppresses the lung and pancreatic tumorigenesis caused by constitutively active K-Ras^G12D^ expression [6]. We determined the effect of CncC depletion in combination with Ras^V12^ expression in the PG on the time of pupation and on pupal size. When the shRNA targeting CncC was co-expressed with Ras^V12^ in the PG, the premature pupation was suppressed and the pupae were restored to nearly normal size (Figure 5A, 5B). CncC depletion in the PG not only suppressed premature pupation caused by Ras^V12^ expression, but delayed pupation relative to wild type larvae, suggesting that CncC was required for both ectopic and endogenous Ras signaling.

We further examined if CncC depletion affected the consequences of constitutive Ras signaling for pupal development. Most of the animals that expressed Ras^V12^ alone arrested at early pupal stages with no detectable eye pigmentation or wings (Figure 5C, Figure S5A). In contrast, co-expression of the shRNA targeting CncC with Ras^V12^ enabled a majority of the pupae to develop to late stages, and some to eclose and produce adult flies (Figure 5C, Figure S5A). It is unlikely that CncC depletion affected Ras^V12^ expression in the PG since CncC depletion did not alter the level of *ras^V12^* transcripts in salivary glands (Figure 5E). The genetic interactions between CncC depletion and Ras^V12^ expression suggest that CncC mediated the regulation of pupation by the Ras signaling pathway.

To determine if Ras signaling affected CncC binding to chromatin, we investigated if Ras^V12^ expression in salivary glands affected endogenous CncC occupancy on polytene chromosomes. Ras^V12^ expression increased both the number of loci occupied by CncC and the level of CncC occupancy at most loci, but did not affect the level of *cncC* transcripts in salivary glands (Figure 5D, Figure S5B). Ras^V12^ expression reduced CncC binding at some loci (Figure 5D). Ras signaling therefore regulated both the efficiency and the specificity of CncC binding to chromatin.

To establish if Ras signaling and CncC affected gene transcription in concert, we examined the effects of ectopic Ras^V12^ and CncC expression on transcription of genes at two of the loci where Ras^V12^ expression affected CncC occupancy in salivary glands (Figure 5D, lower panels). Both Ras^V12^ as well as CncC fusion protein expression activated transcription of these genes (Figure 5E). Conversely, CncC depletion by shRNA expression counteracted the activation of these genes by Ras^V12^ expression. Ras^V12^ expression had selective effects on the transcription of genes at these loci since the transcription of other CncC target genes, including *gstD1* and *gstE1*, was not detectably affected by Ras^V12^ expression (Figure 5E). These results suggest that Ras signaling regulated CncC transcriptional activity by altering its occupancy at selected target genes.

## Discussion

Visualization of the subcellular distributions of CncC and dKeap1 and their occupancy on polytene chromosomes revealed that both CncC and dKeap1 were predominantly nuclear and occupied specific chromatin loci. The nuclear localization of dKeap1 and its occupancy of specific chromatin loci indicated that it has functions distinct from those that have been previously attributed to mammalian Keap1. Analysis of the transcriptional and developmental consequences of tissue-specific depletion of CncC and dKeap1 as well as of mutations in *cncC* and *dKeap1* established that these proteins control transcriptional regulons in different organs that coordinate the onset of metamorphosis. The direct roles of CncC and dKeap1 both in ecdysone biosynthetic gene transcription in the PG as well as in ecdysone-regulated gene transcription in salivary glands establish mechanistic links between these central processes in *Drosophila* metamorphosis.

### Concordant versus opposing effects of CncC and dKeap1 on transcription of different classes of genes

Both CncC and dKeap1 depletion reduced transcription of ecdysone-regulated early puff genes. These loci were occupied by both CncC and dKeap1, suggesting that CncC and dKeap1 activated transcription of these genes in concert. Similarly, transcription of most ecdysone biosynthetic genes was reduced by both CncC and dKeap1 depletion as well as by the *cnc^K6/K6^* and *dKeap1^EY5/EY5^* loss of function mutations in larvae and embryos, respectively. The ecdysone biosynthetic genes were also occupied by both CncC and dKeap1 in embryos, suggesting that CncC and dKeap1 activated their transcription in concert. In contrast, CncC and dKeap1 depletion as well as the *cnc^K6/K6^* and *dKeap1^EY5/EY5^* mutations had opposite effects on transcription of the *gstD1* and *gstE1* xenobiotic response genes in salivary glands and in embryos, respectively. Similarly, opposite effects of CncC and dKeap1 on transcription of other xenobiotic response genes have been previously reported in adult *Drosophila* [2]. CncC and dKeap1 therefore regulated transcription of different classes of genes through distinct mechanisms. Whereas xenobiotic response genes are regulated by antagonistic effects of dKeap1 on transcription activation by CncC, ecdysone biosynthetic and response genes were activated by concerted chromatin binding by CncC and dKeap1. Chromatin binding by dKeap1 as well as its cooccupancy and cooperation with CncC have potential implications for Keap1 function and its effects on Nrf2 activity in mammalian cells. Keap1 can shuttle into the nucleus in some cells [10], [12], and could bind chromatin in association with Nrf2 or other interaction partners.

### CncC and dKeap1 regulate the onset of metamorphosis by controlling transcriptional regulons in different organs

**Figure 6:**
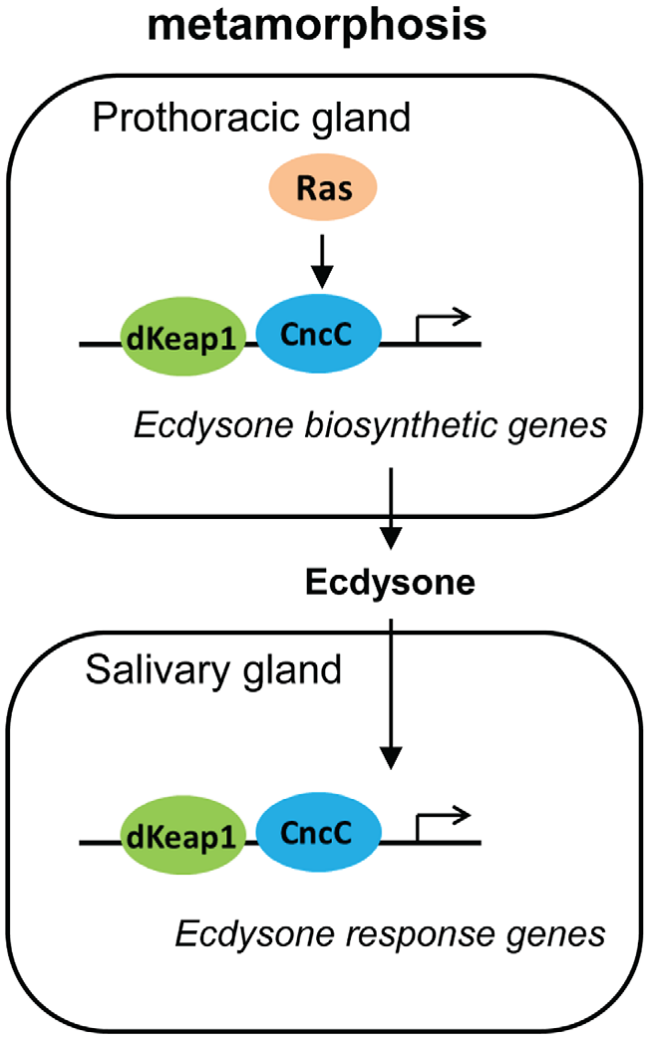
Model for the regulation of the onset of metamorphosis by CncC and dKeap1. The roles of Ras signaling and ecdysone biosynthesis in the control of metamorphosis were previously established [17], [18], [19]. Our results show that CncC and dKeap1 regulate ecdysone biosynthetic genes and that CncC links Ras signaling to ecdysone biosynthetic gene transcription in the PG. The consequent modulation of ecdysone biosynthesis can regulate the onset of metamorphosis. CncC and dKeap1 also regulate the transcription of ecdysone-inducible genes in the salivary gland. We propose that CncC and dKeap1 coordinate the developmental programs that regulate the onset of metamorphosis by controlling both ecdysone biosynthetic and response genes in different tissues.

The effects of CncC and dKeap1 depletion on ecdysone biosynthetic gene transcription and on the timing of pupation indicate that CncC and dKeap1 are important components of the transcription regulatory circuit that controls ecdysone biosynthesis (Figure 6). Many parts of the neuro-endocrine signaling axis that induces ecdysone biosynthesis have been characterized [19], [20], [30], [31]. Previous studies had not identified the transcription factors that bind and regulate ecdysone biosynthetic genes. dSmad2 depletion in the PG reduces ecdysone biosynthetic gene transcription and inhibits pupation. dSmad2 depletion also reduces *torso* and *InR* transcription, and Ras^V12^ or InR co-expression in combination with dSmad2 depletion restores both ecdysone-biosynthetic gene transcription as well as pupation [30]. It is therefore likely that dSmad2 affects ecdysone production indirectly by altering Torso or Insulin signaling. In contrast, CncC depletion suppressed the premature pupation caused by Ras^V12^ expression in the PG, and Ras^V12^ expression in salivary glands altered the loci occupied by CncC on polytene chromosomes. These results, together with CncC occupancy and regulation of ecdysone biosynthetic genes in embryos, suggest that CncC mediated the effects of Ras signaling in the PG on pupation by regulating ecdysone biosynthetic gene transcription.

CncC and dKeap1 also regulated transcription of the early ecdysone-inducible genes in the salivary gland. CncC and dKeap1 binding at ecdysone-inducible early puffs, and the absence of effects of CncC depletion on ecdysone receptor subunit or on late puff gene transcription indicate that CncC and dKeap1 regulated early puff gene transcription directly. The functions of CncC and dKeap1 in regulation of genes that control both ecdysteroid synthesis as well as the transcriptional responses to this hormone place CncC-dKeap1 complex at the nexus of a regulatory network that that coordinates the onset of insect metamorphosis (Figure 6).

### Relationships between xenobiotic responses and *Drosophila* development

The discovery that CncC and dKeap1 coordinate *Drosophila* metamorphosis has identified novel functions of Nrf2-Keap1 family proteins in normal cellular processes and development. The regulation of both metamorphosis and xenobiotic responses by CncC and dKeap1 suggests that these processes either share a common evolutionary ancestry, or that they are mechanistically or functionally interrelated. Most of the ecdysone biosynthetic genes encode cytochrome P450 class oxidoreductases [18]. P450 class oxidoreductases are also key mediators of the metabolic detoxification of many xenobiotic compounds [32].

The genes that were regulated by CncC and dKeap1 in the salivary and prothoracic glands during larval development and those that are regulated by CncC and dKeap1 in adult flies [2], [3] were mostly non-overlapping. Among the genes identified in this study, only *nvd* among the ecdysone biosynthetic genes and *Sgs5* among the ecdysone-regulated genes were detected by microarray analysis of transcripts induced by CncC expression in adult flies [2]. Thus, the effects of CncC and dKeap1 on the transcription of most of the genes that controlled the onset of metamorphosis were restricted to specific tissues and stages of development.

The functions of CncC and dKeap1 in both hormonal regulation of development and in responses to toxic compounds and environmental stress could represent a mechanism that controls development in response to environmental conditions. Imaginal disc damage inhibits PTTH synthesis, resulting in reduced ecdysone synthesis and a delayed pupation [33]. Modulation of TOR signaling in the PG regulates ecdysone biosynthetic and ecdysone-regulated gene transcription and the timing of pupation [31]. Activation of TOR signaling in the PG suppressed the pupation delay caused by larval starvation, indicating that TOR signaling affected developmental timing in response to nutrient stress. Nutrient restriction and heat stress alter 20E and juvenile hormone levels in the ovaries, arresting oogenesis [34]. The interaction between CncC and dKeap1 could mediate responses to both external as well as endogenous signals that modulate developmental progression. Future studies of the effects of environmental stresses on the developmental functions of CncC and dKeap1 will test this hypothesis.

### Regulation of Nrf/CncC family protein functions by Ras signaling

The premature pupation and developmental arrest caused by constitutively active Ras^V12^ expression were suppressed by CncC depletion in the PG. Similarly, the lung and pancreatic tumorigenesis caused by constitutive K-Ras^G12D^ expression are suppressed by Nrf2 deletion in mice [6]. K-Ras^G12D^ expression can cause a two-fold increase in Nrf2 transcription, but the significance of this change in Nrf2 transcription for tumorigenesis has not been established. Ras^V12^ expression in *Drosophila* did not alter the level of CncC transcription, but increased the overall level of CncC binding to chromatin, shifted the loci occupied by CncC on polytene chromosomes, and activated genes at those loci in concert with CncC. These results suggest that Ras signaling can regulate the functions of CncC/Nrf family proteins by altering their target gene specificities or transcriptional activities. The mechanisms whereby Ras regulated CncC occupancy remain to be determined, but are likely to include phosphorylation as the MAPK pathway has been proposed to regulate both Nrf2 and the *C. elegans* homologue of CncC [35], [36], [37].

### Relationships between the functions of CncC and mammalian Nrf family proteins

The relationships between the roles of CncC and dKeap1 in *Drosophila* metamorphosis and the functions of their mammalian homologues in development remain to be elucidated. Two of the mammalian homologues of CncC, Nrf1 and Nrf2, appear to have partially overlapping functions during mouse development [38], [39], [40], [41]. Genome-wide analyses have identified many genes occupied by Nrf2 that have no known functions in the xenobiotic response [42], [43]. Although ecdysteroids are unique to invertebrates, steroid hormones have central roles in many aspects of mammalian physiology. Nrf2 can mediate the 1α,25-dihydroxyvitamin D_3_-induced differentiation of acute myeloid leukemia cells through multiple mechanisms, including VDR/RXRα transcription [44]. Further studies of the mechanisms of action of CncC/Nrf and dKeap1/Keap1 family proteins in different phyla are required to establish the evolutionary relationships among these proteins and their functions in development and disease.

## Materials and Methods

### *Drosophila* stocks

Plasmids encoding CncC, CncB, and dKeap1 fused to intact fluorescent proteins and fluorescent protein fragments were constructed as described in supplemental materials and methods. Transgenic *Drosophila* lines carrying these expression constructs were generated by microinjection in the *w^1118^* background. The transgenic lines carrying *UAS-cncC-RNAi*, *UAS-dKeap1-RNAi* and *UAS-ras^V12^* transgenes were as described [3], [20]. The transgenic line carrying *UAS-cnc-RNAi* expressed an shRNA that targets all of the Cnc. Double transgenic lines were produced by crosses with *Sgs3-GAL4*, *71B-GAL4*, *5015-GAL4*, *phm-GAL4* and *tub-GAL4* driver lines [19], [22], [25], [27]. To minimize external sources of stress, all studies were conducted with larvae and embryos maintained at 25°C with the exception for larvae carrying the *UAS-cnc-RNAi* and *UAS-dKeap1-RNAi* transgenes, which were maintained at 29°C to improve the efficiencies of CncC and dKeap1 depletion. Homozygous and heterozygous embryos carrying the *cnc^K6^* and *dKeap1^EY5^* alleles were identified by using the Dfd-YFP marker.

### Antisera, polytene chromosome squash, immunostaining, and imaging

Anti-CncC and anti-dKeap1 antisera were raised against proteins encompassing residues 88–344 of CncC and residues 620–776 of dKeap1 fused to GST. The antigens were immobilized and used for affinity purification of the antibodies. Polytene chromosome spreads isolated from the salivary glands of early wandering 3^rd^ instar larvae were prepared and immunolabeled as described in supplemental information. Salivary glands, brain complexes (including brain and prothoracic gland), and imaginal discs were isolated from early wandering 3^rd^ instar larvae and were immunolabeled as described in supplemental information.

### Transcript quantitation and chromatin immunoprecipitation

mRNA was isolated from the salivary glands and brain complexes of early wandering 3^rd^ instar larvae as well as embryos, and was quantified by RT-qPCR. The relative transcript levels were calculated by assuming that they were proportional to 2^−Cp^, and were normalized by the levels of *Rp49* transcripts. For ChIP analysis, chromatin was isolated from dechorionated embryos, sheared by sonication, and precipitated using the antisera indicated. The precipitated DNA was quantified by qPCR.

### Analysis of the time of pupation and 20E levels

Newly molted 3^rd^ instar larvae or newly hatched 1^st^ instar larvae were collected and transferred into vials. The number of white prepupae (WPP) was scored every 12 hours or 24 hours. To determine the effect of 20E feeding on pupation, the larvae were grown on feeding plates topped with yeast paste containing 0.5 mg/ml 20E. 20E was extracted from larvae and white pre-pupae and was quantified using an enzyme immunoassay kit (Cayman Chemical). Detailed experimental procedures and references are included in Text S1, Table S1, Table S2.

## Supporting Information

Figure S1 Subcellular localization of CncC and dKeap1 and the specificities of anti-dKeap1 and anti-CncC antibodies. (A). Subcellular distributions of the dKeap1 and CncC fusion proteins in living salivary gland cells. Salivary glands that expressed rxYFP-CncC or rxYFP-dKeap1 under the control of the *Sgs3-GAL4* driver were imaged within 5 minutes after dissection. Images are representative of the salivary glands of all larvae that expressed the fusion proteins. The scale bars at 10 µm. Results: The CncC fusion protein was mostly nuclear in all cells. The dKeap1 fusion was partially nuclear, with a reticular distribution in the cytoplasm. rxYFP-CncC was expressed at different levels in individual salivary gland cells. In cells that expressed high levels of rxYFP-CncC, reticular cytoplasmic fluorescence was observed. The distributions of CncC and dKeap1 appeared to be similar to each other both in the nucleus and in the cytoplasm. High levels of rxYFP-CncC resulted in an aberrant morphology of salivary gland cells. (B). Visualization of dKeap1 and CncC in the prothoracic gland and in imaginal disc cells. The proteins indicated in the images were visualized in the tissues indicated above the images. Ectopically expressed rxYFP-dKeap1 was visualized by imaging intrinsic fluorescence in freshly dissected tissue (yellow, middle panels). rxYFP-dKeap1 was expressed under the control of the *tubulin-GAL4* driver. Ectopic rxYFP-CncC was visualized by imaging intrinsic fluorescence in freshly dissected tissue (lower left) and endogenous CncC was visualized by immunostaining using anti-CncC antibodies (lower right). rxYFP-CncC was expressed under the control of the *5015-GAL4* driver. Hoechst staining of the nuclei (blue) is shown separately in the monochrome images to the right of each color image. Results: Both endogenous dKeap1 (Figure 1C) and the dKeap1 fusion were present within the nuclei of polyploid prothoracic gland cells and of diploid imaginal disc cells. Likewise, both the CncC fusion and endogenous CncC were predominantly nuclear in prothoracic gland cells and in imaginal disc cells. Thus, CncC and dKeap1 were localized to the nuclei in many different tissues and cell types. (C). Specificity of dKeap1 immunoreactivity. The midgut of control (*w^1118^*) and dKeap1 null (*dKeap1^EY5/EY5^*) early 1^st^ instar larvae were immunostained using anti-dKeap1 antibodies (images to the left). Extracts of early 1^st^ instar larvae of the genotypes indicated above the lanes were analyzed by immunoblotting using anti-dKeap1 and anti-tubulin antibodies as indicate below the blots (immunoblots on the right). The bands corresponding to endogenous dKeap1 are indicated. Results: The intensity of anti-Keap1 immunoreactivity was markedly reduced in *dKeap1^EY5/EY5^* larvae, and the band corresponding to endogenous dKeap1 was not detected by immunoblotting of extracts from these larvae, demonstrating the specificity of the anti-dKeap1 antibodies. (D). Specificity of CncC immunoreactivity. Extracts of early 1^st^ instar larvae of the genotypes indicated above the lanes were analyzed by immunoblotting using anti-CncC and anti-tubulin antibodies as indicate below the blots. The bands corresponding to endogenous CncC are indicated. Results: The band corresponding to endogenous CncC was not detected by immunoblotting of extracts from *cnc^K6/K6^* larvae, demonstrating the specificity of the anti-CncC antibodies.
(EPS)

Figure S2 Effects of CncC depletion on puff gene transcription and on larval ecdysteroid levels. (A). Effects of CncC depletion in larvae produced by two different cncC-RNAi sub-lines on transcription of ecdysone-regulated genes in salivary glands. The levels of the transcripts indicated below the bars were measured in salivary glands that expressed the shRNA targeting CncC under the control of the *71B-GAL4* driver. The transcript levels were measured in larvae produced by two sub-lines that had been propagated separately for more than two years (*71B>cncC-RNAi-1* and *71B>cncC-RNAi-2*, striped and solid bars) and in larvae that carried the *cncC-RNAi* transgene, but lacked a GAL4 driver (open bars). To facilitate comparison of the transcript levels, the level of each transcript was normalized by the level of the transcript in the control larvae (*71B-GAL4* or *w^1118^*). All transcript levels were normalized by the levels of the *Rp49* transcript. The data represent the means and the standard deviations from two separate experiments (*, p<0.05). (B). Effects of CncC depletion in the salivary glands on the level of 20E in the larvae. The levels of 20-hydroxyecdysone (20E) were measured in the salivary glands of early wandering third instar larvae of control larvae (*71B>*) and transgenic larvae that expressed the shRNA targeting CncC under the control of the *71B-GAL4* driver (*71B>cncC-RNAi*). The data represent the means and standard deviations from two repeats using 10 larvae in each. Results: We considered the possibility that the effects of CncC depletion on ecdysone-regulated gene transcription were caused by changes in ecdysteroid levels in the larvae. CncC depletion in the salivary glands had no detectable effect on the level of 20E in the larvae.
(EPS)

Figure S3 Effects of CncC and dKeap1 depletion in the PG on ecdysone biosynthetic gene transcription and PG morphology. CncC and dKeap1 occupancy at the *sad* gene. (A). Effects of CncC depletion on the transcription of ecdysone biosynthetic genes in prothoracic glands. The levels of the transcripts indicated above the upper graphs were measured in the brain complexes from control larvae (*5015>*, open bar) and from larvae produced by two sub-lines expressing the shRNA targeting CncC under the control of the *5015-GAL4* driver (*5015>cncC-RNAi-1* and *5015>cncC-RNAi-2*, striped and solid bar). The data represent the means and standard deviations of two separate experiments (*, p<0.05). Results: The same changes in ecdysone biosynthetic gene transcription in the PG were observed in both *cncC-RNAi* sub-lines, demonstrating the genetic stability and reproducibility of these effects. (B). Effects of CncC depletion in the PG on Sad protein expression. The dissected brain complexes of control larvae (*phm>*) and larvae that expressed the shRNA targeting CncC in the PG (*phm>cncC-RNAi*) were stained with anti-Sad antibody (red) and Hoechst (cyan). Results: Expression of the shRNA targeting CncC in PG under the control of *phm-GAL4* driver reduced Sad immunoreactivity in the PG. The polyploid nuclei of the PG were identified based on their large size compared to the diploid nuclei of the brain (right panels). The size and the number of nuclei in the PG were not altered by expression of the shRNA targeting CncC, suggesting that CncC depletion did not disrupt the overall structure of the PG (see also Figure 3C). (C). Effects of dKeap1 depletion on PG morphology. The dissected brain complexes of larvae that expressed the shRNA targeting dKeap1 in the PG were stained using Hoechst. Results: The size and the number of nuclei in the PG were not altered by expression of the shRNA targeting dKeap1, suggesting that dKeap1 depletion did not disrupt the overall structure of the PG. (D). CncC and dKeap1 occupancy of different regions of the sad gene and of the promoter regions of other ecdysone biosynthetic genes in late embryos. Chromatin isolated from stage 14–16 embryos was precipitated using anti-dKeap1 (striped bar), anti-CncC (solid bar) or pre-immune (open bar) sera. The promoter regions of the genes indicated below the bars, and regions at different distances from the *sad* transcription start site as indicated below the bars were quantified using qPCR. The data represent the mean values and standard deviations of replicate qPCR reactions, and are representative of two independent experiments. Results: CncC and dKeap1 were enriched near the transcription start site of the sad gene as well as at the promoter regions of other ecdysone biosynthetic genes.
(EPS)

Figure S4 Effects of CncC depletion in the PG on pupation and ecdysteroid level. (A). Expression of the shRNA targeting CncC in the PG produces giant semi-pupae. Comparison of the pupae formed by control larvae (*phm>*) and semi-pupae formed by larvae that expressed the shRNA targeting CncC under the control of the *phm-GAL4* driver (*phm>cncC-RNAi*) 7 days after 3^rd^ instar ecdysis. Results: A small proportion (2–5%) of the larvae that expressed the shRNA targeting CncC in the PG did not fully pupate within 7 days after 3^rd^ instar ecdysis and formed giant semi-pupae. (B). Effects of expression of the shRNA targeting CncC in the PG on 20E biosynthesis. The levels of 20E were measured in early wandering control larvae (*5015>*) and larvae that expressed the shRNA targeting CncC in the PG (*5015>cncC-RNAi-1* and *5015>cncC-RNAi-2*) 60 hours after 3^rd^ instar ecdysis. The data represent the means and standard deviations from two separate experiments with 10 larvae each (*, p<0.05).
(EPS)

Figure S5 Genetic interactions between Ras^V12^ and CncC in the PG. (A). Effects of the expression of Ras^V12^ and the shRNA targeting CncC separately and together in the PG on pupal development. The proportion of pupae that were arrested at early (P1–P9; open bar) and late (P10–P15; striped bar) stages as well as those that eclosed and produced adults (solid bar) were recorded for larvae that expressed either Ras^V12^ alone (*phm>ras^V12^*), Ras^V12^ in combination with the shRNA targeting CncC (*phm>ras^V12^, cncC-RNAi*), or the the shRNA targeting CncC alone (*phm>cncC-RNAi*) in the PG. The proportion of animals in each category was determined in approximately 100 animals of each genotype. Results: To determine the effects of the genetic interaction between the expression of Ras^V12^ and of the shRNA targeting CncC in the PG, we examined their effects on pupal development. When Ras^V12^ was expressed alone under the control of the *phm-GAL4* driver, 93% of the pupae were arrested at early stages with no detectable eye pigmentation or wings. In contrast, the majority (68%) of pupae formed by larvae that expressed Ras^V12^ in combination with the shRNA targeting CncC under the control of the *phm-GAL4* driver developed to late stages with detectable eye pigmentation and wings. The genetic interactions between expression of Ras^V12^ and the shRNA targeting CncC suggest that CncC mediated the regulation of pupation by the Ras signaling pathway. (B). The transcription of cncC was not affected by RasV12 expression in the salivary glands. The level of the transcript was measured in the salivary glands of control larvae (*Sgs3>*, open bar) and of larvae that expressed Ras^V12^ in the salivary glands (*Sgs3>ras^V12^*, solid bar). The transcript level was normalized by the level of the *Rp49* transcript and represents the mean and standard deviation from two separate experiments.
(EPS)

Table S1 Primer sequences used to measure the transcript levels by RT–qPCR.
(PDF)

Table S2 Primer sequences used to measure CncC and dKeap1 occupancy by ChIP.
(PDF)

Text S1 Supporting Materials, Methods, and References. Plasmid expression vectors. *Drosophila* stocks. Antisera, polytene chromosome squash, immunostaining and imaging. Immunoblotting. Quantitation of transcript levels. Chromatin immunoprecipitation (ChIP) analysis. Analysis of the time of pupation. Measurement of 20E levels. Statistical analyses.
(PDF)
