# Supplementary material for: Regulation of Drosophila Metamorphosis by Xenobiotic Response Regulators
Source: PLoS Genet. 2013 Feb 7;9(2):e1003263. doi: 10.1371/journal.pgen.1003263 (PMC3567155; doi:10.1371/journal.pgen.1003263)
Supplement: Table S1 — Primer sequences used to measure the transcript levels by RT–qPCR. (PDF) [file pgen.1003263.s006.pdf]

Table S1. Primer sequences used to measure the transcript levels by RT-qPCR

gene	5'-primer	3'-primer
Rp49	CGGATCGATATGCTAAGCTGT	GCGCTTGTTCGATCCGTA
cncC	GAGGTGGAAATCGGAGATGA	CTGCTTGTAGAGCACCTCAGC
dKeap1	CAAGGAGTCGGAGATGTCG	GTAGAGGATGCGTGACATGG
Sgs1	TCGCCCTTATCTTTTTAACTGTG	CCTCCACATCCGGGTATTATC
Sgs3	ACCATTGCTACCGCCCTA	GCATCCACAATCGCAACA
Sgs4	AGGCAAGAAGAACACCACCA	TTGCTGTTTAGCAACCACCTT
Sgs5	GTGCCACCTGCTGAAATTCT	GAAGGGCCAACAATAGGAATAA
Sgs7	ACTGATCGCAGTCACCATCA	AGGGCTAGATCGGAGAATCC
Sgs8	CAACAACCATGAAGCTGCTC	CGAATCCGATGAGCATGA
BR-C	CTACAACCGCACCATCCAG	ATGCGTTACGATGCGATG
hsp23	TACTTGGCCCTGGTTGGAC	GCCCACCTGTTTCTCCAG
E74	AACATATGGAATCGCAGTGTTG	CAGGTAGCCAGGCGTTTG
E75	CTCCATGCCCACACTTCTG	AATTACGTGGGCGAAACG
E78	GACGGTGCCACAAAATGG	ACCCTGGCCGAGAACTGT
E63-1	ATTGGGGAGCCCTTGAAC	AGCAAAAGTCTCGTGAATTCCT
E23	CGAAGGATCGGCTATGTCC	GCAGTATAGACCACCGTTTCG
ImpL1	CCATACCAAGTAGTTTGCATCGTA	ATCGGAGCCCTTGTGCTT
ImpL2	GCCGATACCTTCGTGTATCC	TTTCCGTCGTCAATCCAATAG
ImpL3	CATCATCCCCAAGCTGGTAG	CCAGGCCACGTAGGTCAT
L63	GCACAGCTTCGGCAGATTA	TGATGGTGGTGGTGATGGT
L82	CGAGGAGATTAGCGACCTGA	AAGAGCGAGGTCTTGCGATA
EcR	TTAATTTGGTACCAGGATGGCTA	GTTCTCATCGGGTTGACTCAT
usp	GCTCCTTTGAGCGACGAT	CGAGAAGCTCTGGTTGAGGA
gstD1	TCGCGAGTTTCACAACAGAA	TGAGCAGCTTCTTGTTCAGC
gstE1	GGACTACGAGTACAAGGAGGTGA	TCACATATTCCTCGCTCAGGT
sad	GATGTGCCAGGCGATATGAT	ACTGCTGAATGCGGTCGT
dib	GTGACCAAGGAGTTCATTAGATTTC	CCAAAGGTAAGCAAACAGGTTAAT
nvd	CGGGGTACAGACGGATTATG	TGTTCAGCATGATACCAAATAAAAA
spok	GCGGTGATCGAAACAACTC	CGAGCTAAATTTCTCCGCTTT
phm	GTGGATTTGGCATCATCTG	CAAGGCCTTTAGCCAATCG
start1	GGGCACATTCCATAAACCTG	GGCAGAAAAACCTTCGAACC
ras	CGAGGACTCTTACCGAAAGC	GGCCGAGTACTCCTCTTGG
Cp1	ACTACACTCTGCACAAGCAACTG	CGGCGAGATGAAGGTGAC
Cyp28c1	CATGCCATGACCTTCATGTT	ATCTGGATTACGACCAAGCAG
