# Supplementary material for: Regulation of Drosophila Metamorphosis by Xenobiotic Response Regulators
Source: PLoS Genet. 2013 Feb 7;9(2):e1003263. doi: 10.1371/journal.pgen.1003263 (PMC3567155; doi:10.1371/journal.pgen.1003263)
Supplement: Table S2 — Primer sequences used to measure CncC and dKeap1 occupancy by ChIP. (PDF) [file pgen.1003263.s007.pdf]

Table S2. Primer sequences used to measure CncC and dKeap1 occupancy by ChIP

gene	position	5’-primer	3’-primer
sad	-5000	GCCAACGTCTGGTCAAAAAT	CCAAGTTCTTTTTGTGCTCTGTT
	-1200	CCCCTAATAGACCATAAATATGACAAG	CTTGACGAAGGTTGTGAAAATG
	60	GAGCAGGTGCAGGTGGTTAC	TCTTCTCGGTCATCGGTGTA
	4100	ATCGCTCATGTTTGCCTTTT	GCTCAAAGTGTGATGGATCG
dib	-100	TTCACCACAGGTAGGCACAA	CATTCGGGAAAAAGGCATTA
nvd	-100	CGCGTATGCCGGAAAAATA	GTTCACCGTTTTCGTTAGGG
spok	300	GTCCGCCAAAGTATTTTCCA	TTACGGTTTTGGCACATTGA
phm	0	CTGATGTTACCATTGTCTGAACG	TCAACGTGTGTCTACGTCAGC
shd	-200	GGATCGATGGAAGCACTTGT	ACGATGGGAGGCGATTTA
dKeap1	700	AAGGTTTTGCCTCTTCAGCA	CGGCTTTAAATTTCCGCATA
gstD1	-1000	CATGTGCCTTTGACTCATCATT	GACCATAAAAATATAACCGTTTTTCG
Rp49	0	TCCTTCCAGCTTCAAGATGAC	GTGCGCTTCTTCACGATCT
Actn3	1200	CGAAGAAACAGCCAAAATCG	GGACGCCACTTTGTATTGAAC
Gapdh1	300	GAAAAAGCGGCAGTCGTAAT	AATTCCGATCTTCGACATGG
