# Supplementary material for: Regulation of Drosophila Metamorphosis by Xenobiotic Response Regulators
Source: PLoS Genet. 2013 Feb 7;9(2):e1003263. doi: 10.1371/journal.pgen.1003263 (PMC3567155; doi:10.1371/journal.pgen.1003263)
Supplement: Text S1 — Supporting Materials, Methods, and References. Plasmid expression vectors. Drosophila stocks. Antisera, polytene chromosome squash, immunostaining and imaging. Immunoblotting. Quantitation of transcript levels. Chromatin immunoprecipitation (ChIP) analysis. Analysis of the time of pupation. Measurement of 20E levels. Statistical analyses. (PDF) [file pgen.1003263.s008.pdf]

Supporting Materials and Methods

Plasmid expression vectors

The CncC-rxYFP and dKeap1-rxYFP expression vectors encoded rxYFP fused to the C-termini of CncC and dKeap1, respectively, in pUAST. [1]. rxYFP contained the N149C and S202C substitutions in YFP [2]. The CncC-YN expression vectors encoded residues 1-173 of YFP fused to the C-termini of CncC in pUAST

Drosophila stocks

Drosophila lines carrying the rxYFP-CncC and rxYFP-dKeap1 transgenes were generated by microinjection in the w^{1118} background by BestGene Inc. Lines carrying the CncC-YN transgene was generated by microinjection in the w^{1118} background by Osamu Shimmi. Larvae expressing the CncC and dKeap1 fusion proteins in specific tissues were obtained by crossing the lines carrying expression vectors with the driver lines indicated and were analyzed in the F1 generation. The *CyO*, *TM3Sb*, and *TM2Ubx* balancers were used in the crosses to select appropriate progeny.

Sgs3-GAL4 and *71B-GAL4* driver lines activate transgene expression in salivary glands during late 3rd instar, and in imaginal discs and salivary glands throughout embryonic and larval development, respectively [3,4]. The *5015-GAL4* driver line activates transgene expression in the PG and in salivary glands [5]. The *phm-GAL4* driver line activates transgene expression in the PG and in the wing and leg discs of late 3rd instar larvae [6]. The *tub-GAL4* driver line activates transgene expression in all tissues from embryo to adult [7].

The transgenic lines carrying *UAS-cncC-RNAi* and *UAS-dKeap1-RNAi* were described [8]. The *UAS-cncC-RNAi* and *UAS-dKeap1-RNAi* alleles are homozygous lethal and were maintained with the *CyO, Dfd-YFP* balancer. Transgenic larvae that expressed the shRNA in different tissues were produced by crossing this line with different *GAL4* driver lines and screening the larvae for intrinsic fluorescence produced by the Dfd-YFP marker. The transgenic line carrying *UAS-cnc-RNAi* was generated by the Vienna *Drosophila* RNAi Center (VDRC construct ID 4437) and is predicted to target all Cnc isoforms. The *UAS-ras*^{V12} transgene encodes Ras with the G12V mutation [9]. The *UAS-cncC-RNAi, UAS-ras*^{V12} double transgenic line was generated by crossing the lines containing each allele and was maintained as an *UAS-cncC-RNAi* / *CyO, Dfd-YFP*; *UAS-ras*^{V12} stock. To generate larvae for experiments, this line was crossed with a *GAL4* driver line and the F1 progeny lacking the Dfd-YFP marker were compared with the F1 progeny of *UAS-ras*^{V12} flies crossed with the *GAL4* driver line. *Drosophila* stocks were maintained and all genetic experiments were performed at room temperature (24-26°C), with the exception for the transgenic lines that expressed *cncC-RNAi* targeting all Cnc isoforms and that expressed *UAS-dKeap1-RNAi*, which were cultured at 29°C to enhance the efficiencies of CncC and dKeap1 depletion.

The *cnc*^{K6} allele contains a nonsense mutation at amino acid position 471 that is present in CncC, but not in the CncB or CncA coding regions [10]. The *dKeap1*^{EY5} allele contains a P-element insertion in the 4th exon [8]. *cnc*^{K6} and *dKeap1*^{EY5} were maintained as heterozygous stocks carrying a *TM6,Tb,Sb,Dfd-YFP* balancer chromosome. Embryos that were homozygous or heterozygous for

cnc^{K6} or *dKeap1*^{EY5} were collected from F1 progenies of heterozygotes and were identified based on the intrinsic fluorescence produced by the Dfd-YFP marker. All previously described lines were obtained from the Bloomington Stock Center, with the exception for *phm-GAL4* (Michael O'Connor), *UAS-cncC-RNAi* (Dirk Bohmann and Osamu Shimmi), *UAS-dKeap1-RNAi*, *dKeap1*^{EY5} (Dirk Bohmann), *cnc*^{K6} (Osamu Shimmi and William McGinnis), and *UAS-cnc-RNAi* (Vienna *Drosophila* RNAi Center).

Antisera, polytene chromosome squash, immunostaining and imaging

Anti-CncC and anti-dKeap1 antisera were raised against proteins encompassing residues 88-344 of CncC and residues 620-776 of dKeap1 fused to GST. The antisera were affinity purified by incubating them with CncC or dKeap1 fusion proteins bound to nitrocellulose membranes, followed by elution. Anti-GFP antibody (Fitzgerald) and goat anti-rabbit conjugated to Alexa Fluor 594 (Invitrogen) were used.

Polytene chromosome squashes were prepared by dissecting 2-3 pairs of salivary glands and fixing them in 200 µl PBS + 4% paraformaldehyde + 1% Triton X-100 for 1 minute followed by incubation in 200 µl 45% acetic acid + 4% paraformaldehyde for 2 minutes. The fixed salivary glands were transferred to 10 µl 16.7% lactoacetic acid + 25% acetic acid and squashed between a coverslip coated with Sigmacote (Sigma) and a microscope slide coated with poly-lysine (Sigma) as described in detail in Johansen et al. (2009). The polytene chromosome squashes were immuno-labeled using the antibodies indicated as described [11,12].

Immuno-labeling of whole salivary glands, the brain complex (including brain and prothoracic gland), imaginal discs, and midgut were performed as described [13]. Antibodies were diluted as follows: anti-GFP (Fitzgerald Industries Intl.) (1:200), anti-CncC (1:100), anti-dKeap1 (1:100), anti-Lamin Dm0 (ADL67.10, Developmental Studies Hybridoma Bank) (1:1000), anti-Sad (Abcam) (1:200). Alexa Fluor 594 conjugated goat anti-rabbit (Invitrogen) (1:1000). DNA was visualized by staining with Hoechst 33258 (Molecular Probes) in PBS. The samples were mounted in VectaShield (Vector Laboratories) and were imaged using an Olympus IX81 DSU microscope with a Hamamatsu ORCA-ER CCD camera. For live imaging, tissues were dissected, mounted in PBS and imaged within 5 minutes after dissection. rxYFP signal was visualized using 504 nm excitation and 542 nm emission wavelengths. The brightness of images was adjusted by linear scaling of image contrast, the images were pseudo-colored and merged using image processing software.

Immunoblotting

5 pairs of salivary glands dissected in ice-cold PBS from early wandering 3rd instar larvae, or 20 whole early 1st instar larvae, were homogenized in 30 □l ice-cold IP Buffer (20 mM Tris-HCl pH8.0, 0.2% NP-40, 0.2% Triton X-100, 150 mM NaCl, 5 mM EDTA, 1 mM EGTA, 2 mM $NaVO_3$, protease inhibitor cocktail (Roche) and 1 mM PMSF). The samples were resolved using a NuPAGE 4-12% Bis-Tris gel (Invitrogen). The proteins were transferred to Nitrocellulose membrane (Bio-Rad) and probed using CncC antiserum (1:500), dKeap1 antiserum (1:500) or anti-α-tubulin

antibody (12G10, Developmental Studies Hybridoma Bank) (1:500), and HRP-conjugated secondary antibodies (GE healthcare UK limited). HRP activity was detected using ECL Plus and Hyperfilm (Amersham).

Quantitation of transcript levels

10-15 pairs of salivary glands or 20 brain complexes from early wandering 3rd instar larvae were dissected in PBS prepared using DEPC water. mRNA was isolated using the RNeasy kit (Qiagen) , treated with RQ1 RNase-Free DNase (Promega), and reverse transcribed using the Transcriptor First Strand cDNA Synthesis Kit (Roche). Real-time qPCR was performed using SYBR Green I Master (Roche) in a LightCycler 480 II (Roche). The relative transcript levels were calculated by assuming that they were proportional to 2^{-Cp}, and were normalized by the levels of *Rp49* transcripts. Primer sequences were designed using Universal ProbeLibrary software (Roche) and are listed in Table S1.

To collect embryos, heterozygous male and female flies were placed in a fly cage and were allowed to lay eggs on apple juice agar plates over 2 hours. Stage 14-16 embryos (~15 hours) were screened for YFP fluorescence to distinguish homozygotes from heterozygotes. About 100 embryos were collected for mRNA extraction and RT-qPCR analysis of transcript levels as described above.

Chromatin immunoprecipitation (ChIP) analysis

Embryo ChIP samples were prepared using a protocol modified from [14]. About 1 g of embryos were collected using apple juice agar plates, dechorionated with 50% bleach and washed with PBST (phosphate buffered saline + 0.01% Triton X-100). The embryos were transferred to a 50 ml falcon tube and resuspended in 10 ml freshly prepared crosslinking solution (1 mM EDTA, 0.5 mM EGTA, 100 mM NaCl, 1.8% formaldehyde, 50 mM HEPES pH8.0). The suspension was immediately mixed with 30 ml heptane and was vigorously shaken at 25°C for 15 minutes. The organic and aqueous layers were allowed to separate and were carefully aspirated, leaving the embryos. 30 ml stop solution (PBST + 125 mM glycine) was added and the suspension was shaken for an additional 2 minutes. The cross-linked embryos were washed and re-suspended in 5 ml cold PBST containing a cocktail of protease inhibitors (Roche) and 1 mM PMSF. The embryos were homogenized using 7 ml Wheaton Dounce homogenizer with loose pestle, followed by centrifugation at 1100g for 10 minutes in 4°C to pellet the cells. The cells were re-suspended in 5 ml cell lysis buffer (85 mM KCl, 0.5% NP-40, 5 mM HEPES pH 8.0) containing protease inhibitors and PMSF and Dounce homogenized with tight pestle, followed by centrifugation at 2000g for 5 minutes in 4°C to pellet the nuclei. The nuclei were re-suspended in 1 ml nuclear lysis buffer (10 mM EDTA, 0.5% N-lauroylsarcosine, 5 mM HEPES pH 8.0) containing protease inhibitors and PMSF and incubated 20 minutes on ice. The chromatin was sheared by sonication using Diagenode Bioruptor. The chromatin was immunoprecipitated using anti-CncC antiserum, anti-dKeap1 antiserum, or pre-immune serum as described [15]. The precipitated DNA was analyzed by real-time qPCR using the primes listed in Table S2.

Analysis of the time of pupation

Eggs were collected on apple juice agar plates over a 2 hour period. The newly hatched 1st instar larvae were collected and cultured in feeding plates (cornmeal food in 35 mm × 10 mm Corning cell culture dish). Newly molted 3rd instar larvae were collected and transferred into vials (25 mm diameter) with standard cornmeal food. 20 larvae were placed in each vial to avoid overcrowding. The number of white prepupa (WPP) was scored every 12 hours. To determine the effect of 20E feeding on pupation, newly molted 3rd instar larvae were cultured on feeding plates containing 0.3 mg/ml 20E and topped with yeast paste containing 0.5 mg/ml 20E. The 20E (Sigma) stock solution was prepared by dissolving 20E in 95% ethanol at 10 mg/ml. Food for control larvae was prepared using an equal volume of the 95% ethanol vehicle.

Measurement of 20E levels

10 larvae or white pre-pupae were collected at specific times after 3rd instar molting and homogenized in 0.5 ml methanol. The extract was centrifuged at 20000g for 10 min. The precipitate was resuspended in 0.5 ml methanol and sonicated for 15 minutes using a Diagenode Bioruptor sonicator, then re-centrifuged at 20000g for 10 min. The supernatants were combined and evaporated using a vacuum evaporator. The dry residue was dissolved in EIA buffer and 20E levels were measured using an enzyme immunoassay kit with AChE Tracer (Cayman Chemical). Pure 20E (Sigma) was used as a calibration standard.

Statistical analyses

The relative transcript levels measured by RT-qPCR were compared using two-way ANOVA with replicates. The 20E levels and pupa sizes were compared using paired Student's t-tests.

Supporting References

1. Brand AH, Perrimon N (1993) Targeted gene expression as a means of altering cell fates and generating dominant phenotypes. Development 118: 401-415.
2. Ostergaard H, Henriksen A, Hansen FG, Winther JR (2001) Shedding light on disulfide bond formation: engineering a redox switch in green fluorescent protein. EMBO J 20: 5853-5862.
3. Cherbas L, Hu X, Zhimulev I, Belyaeva E, Cherbas P (2003) EcR isoforms in Drosophila: testing tissue-specific requirements by targeted blockade and rescue. Development 130: 271-284.
4. Busson D, Pret AM (2007) GAL4/UAS targeted gene expression for studying Drosophila Hedgehog signaling. Methods Mol Biol 397: 161-201.
5. Yoshiyama T, Namiki T, Mita K, Kataoka H, Niwa R (2006) Neverland is an evolutionally conserved Rieske-domain protein that is essential for ecdysone synthesis and insect growth. Development 133: 2565-2574.
6. Mirth C, Truman JW, Riddiford LM (2005) The role of the prothoracic gland in determining critical weight for metamorphosis in Drosophila melanogaster. Curr Biol 15: 1796-1807.
7. Lee T, Luo L (1999) Mosaic analysis with a repressible cell marker for studies of gene function in neuronal morphogenesis. Neuron 22: 451-461.
8. Sykiotis GP, Bohmann D (2008) Keap1/Nrf2 signaling regulates oxidative stress tolerance and lifespan in Drosophila. Dev Cell 14: 76-85.
9. Lee T, Feig L, Montell DJ (1996) Two distinct roles for Ras in a developmentally regulated cell migration. Development 122: 409-418.
10. Veraksa A, McGinnis N, Li X, Mohler J, McGinnis W (2000) Cap 'n' collar B cooperates with a small Maf subunit to specify pharyngeal development and suppress deformed homeotic function in the Drosophila head. Development 127: 4023-4037.
11. Silver LM, Elgin SC (1976) A method for determination of the in situ distribution of chromosomal proteins. Proc Natl Acad Sci U S A 73: 423-427.
12. Johansen KM, Cai W, Deng H, Bao X, Zhang W, et al. (2009) Polytene chromosome squash methods for studying transcription and epigenetic chromatin modification in Drosophila using antibodies. Methods 48: 387-397.
13. Phalle Bde S (2004) Immunostaining of whole-mount imaginal discs. Methods Mol Biol 247: 373-387.
14. Sandmann T, Jakobsen JS, Furlong EE (2006) ChIP-on-chip protocol for genome-wide analysis of transcription factor binding in Drosophila melanogaster embryos. Nat Protoc 1: 2839-2855.
15. Ren X, Vincenz C, Kerppola TK (2008) Changes in the distributions and dynamics of polycomb repressive complexes during embryonic stem cell differentiation. Mol Cell Biol 28: 2884-2895.
